# The Chinese University stakeholder satisfaction survey: Developing a customer-centered self-assessment tool for higher education quality management

**DOI:** 10.3389/fpsyg.2022.1043417

**Published:** 2022-12-01

**Authors:** Feng Pan, Liu Liu, Zhen Wang

**Affiliations:** ^1^School of Physical Education, Sichuan University, Chengdu, China; ^2^School of Physical Education, National Huaqiao University, Quanzhou, China

**Keywords:** customer-centered, universities, innovative management tool, satisfaction, China

## Abstract

**Introduction:**

Customer-centered management theory has considerable potential for increasing the quality of higher education (HE) in China and promoting its sustainable development.

**Methods:**

This study applied customer-centered enterprise management theory to develop an HE stakeholder satisfaction scale based on data from 1,654 students, teachers, and other staff members, including human resources personnel.

**Results:**

The three-part stakeholder satisfaction survey consists of the China University Student Satisfaction Scale, the China University Teacher and Staff Satisfaction Scale, and the China University Graduate Human Resources Department Satisfaction Scale. All three subscales were valid, reliable, and can be used to foster management innovation, although they require adjustments to improve their coverage of different HE environments.

**Discussion:**

Organizational self-assessment based on customer-centered corporate management theory has much to contribute to the quality and sustainability of China’s HE systems.

## Introduction

In the early 21st century, the rapid growth of new universities in China’s HE sector has led to a renewed focus on the quality of the services they provide. This process has resembled similar shifts that occurred in the European HE sector between the 1950s and 1980s, in which self-assessment was used to secure funding and became a crucial means of guaranteeing teaching quality and promoting institutional development (Peter, 1997). The central argument of this paper is that the internal management of Chinese universities requires similar alterations to raise the quality of provision and that these should be informed by customer-centered contemporary corporate quality management theories, as successfully used in Europe and the United States (Cardona and Bravo, 2012; Sen et al., 2012). As numerous studies attest, self-assessment proactively improves the performance of HE organizations. For instance, Hides et al. (2004) showed that self-assessment based on the “European Quality Award,” model actively influenced the organization’s ability to respond to pressure from its board of directors and other stakeholders while nurturing a customer-centered culture. Ruben et al. (2007) demonstrated that self-assessment in HE supports the acquisition of knowledge and identifies organizational strengths and weaknesses, increasing employees’ commitment to their organization and promoting beneficial change.

Effective corporate self-assessment depends on robust and comprehensive stakeholder satisfaction surveys. In the context of HE, the key stakeholders are teachers, students, and human resources (HR) departments. The current paper describes how a satisfaction survey was developed and tested for each stakeholder type to produce a comprehensive research instrument that can be used to nurture the scientific and sustainable development of internal management in universities in China.

Customer satisfaction can be understood as a person’s emotional response to a product or service (Kotler and Koller, 2016). It is a critical concept in marketing and key to consumer purchasing decisions and behaviors (Sun, 2009). In many countries and regions, consumer satisfaction indices are now widely used to assess quality and macroeconomic development (Fei et al., 2016). The famous ISO9000 quality management system standard proposed a new approach to quality control that placed customer satisfaction at its core: “Make consumers the focus and pursue consumer satisfaction.” Consumer satisfaction has become the starting point and destination for quality strategies in the 21st century (Nan and Zhao, 2008).

Nowadays, consumer satisfaction surveys are widely used in internal HE assessments under the assumption that higher education is a service (Ostrom et al., 2011) with internal and external customers. Internal clients work to accommodate external clients (Muchira and Bett, 2018), i.e., the purchasers or recipients of products or services outside an organization. Internal customers are the individuals or organizations within the corporation itself that receive products or services and whose outputs are linked to the work or actions of other departments or individuals within the same corporation (Maguad, 2007).

The idea of customer satisfaction self-assessment had become widespread in HE in Europe and the US by the mid-to-late 1990s. Researchers believed that the customer satisfaction orientation of such organizations had raised their efficiency to extremely high levels. Indeed, the increasing commercial success of universities has since confirmed that an emphasis on customers is conducive to organizational development (Eurico et al., 2013). Correspondingly, the satisfaction levels of students, teachers, and employers are critical self-assessment indices in almost all universities.

The perspective that students are customers is controversial and many people continue to reject it. However, Guilbault (2018) used a framework of market/customer orientation and service/relationship marketing and believed that students should be considered customers in the development of marketing strategy. Hanover Research (2015) stated that “Universities are recognizing that students are also customers and that excellent customer experiences are required across the student lifecycle.” Nearly all university managers surveyed by Pitman preferred to view students as customers while all respondents surveyed by Maguad (2007) agreed that students were customers, although a few objected to this description. Overseas researchers have found students to be particularly critical internal customers of HE services (Byrnes et al., 1994). They wish to learn skills, obtain knowledge, understand the world around them better, and gain confidence and opportunities in a pleasant environment (Zhang, 2012). If universities meet such expectations, customer satisfaction tends to be high.

**FIGURE 1:**
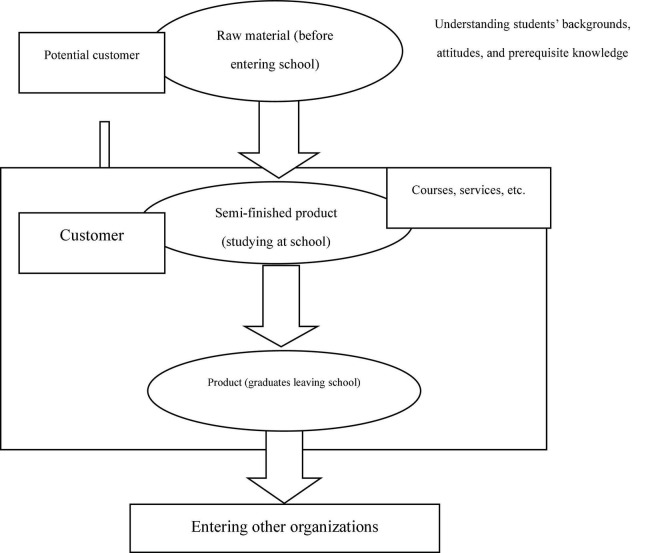
Students’ growth and role based on the quality control of higher education self-assessment (Liu, 2018).

However, if the education system only considers students as customers and overlooks the missions of organizations, the core philosophy underpinning self-assessment is seriously compromised. Viewing students only as internal or external customers creates problems such as the one-sided accommodation of student demands and disregard for the value and function of education. This may impact the positive effects of education on students and their social responsibilities after graduating from university. After students graduate, enter society, and take on jobs, their roles change: they become employees and, as graduates whose talents have been cultivated over several years, the *products* of higher education (Zhang, 2012). This dual character must be recognized in universities’ self-assessment processes: an HE student is not merely an internal or external client but a unique type of customer (see Figure 1).

Co-workers are internal customers who should serve external clients with speed, courtesy, and professionalism to maintain cash flows (Coscia, 2019) and improve corporate development. Teachers, meanwhile, are the pillars of any nation’s education system and are vital to the development of its students. They therefore play a pivotal role in improving the quality of educational institutions (Mansur, 2020). Kolara et al. (2018) argued for the transparency of customer- and market-oriented strategies, suggesting that educational organizations base key performance indicators on internal quality and employee satisfaction. Thus, modern management concepts and viewing teachers as an organization’s internal customers are noteworthy aspects of HE self-assessment processes. Indeed, it is not difficult to find examples of teachers acting as internal customers of universities: they use various facilities such as the university’s library, offices, laboratories, gym, and swimming pool, and they require the school’s administrative departments to provide various services such as HR, transportation, and logistics (Chen and Si, 2006).

The major task of modern HE management is to align organizational and individual goals. The existence and development of a school depend on the labor of teachers, who in turn rely on specific organizational departments to function effectively. Universities and teachers have mutual goals around which management can integrate effective processes. A focus on internal customer demands and satisfaction can encourage teachers to act as organizational members and motivate them to create and maintain competitiveness (Mohsen et al., 2019).

Employers consume the talents cultivated in universities; specifically, they are the direct external customers of the products of higher education (Sirvanci, 1996). Here, “employers” would include institutes where graduates continue their studies or science and research institutions. Rinehart (1993) maintained that service and manufacturing industries and non-profit organizations are the most direct and largest external customers of universities. Every employer hopes that high-quality HE graduates will work efficiently for them and will favor candidates from a particular university based on their previous experience with its alumni. This requires employment-oriented HE institutions—especially vocational schools—to survey levels of employer satisfaction, which is one aspect of a comprehensive quality control process.

Because university graduates increasingly opt to start their own businesses, HE institutions have shifted focus to facilitating high-quality social and technological innovation *via* comprehensive startup ecosystems in universities, cities, and regions (De Jager et al., 2017; Wu et al., 2021). This means that graduates are also likely to become employers, adding to their uniqueness as internal and external customers.

**FIGURE 2:**
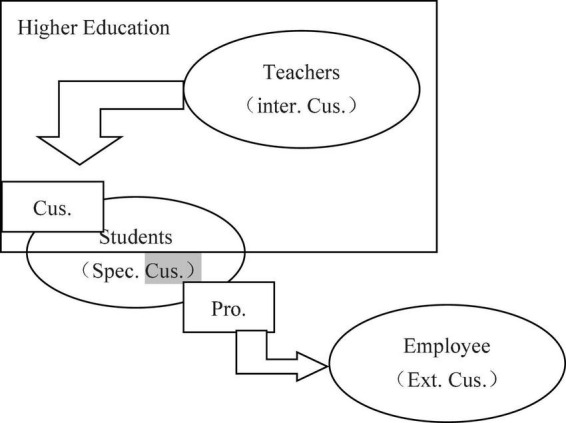
Different customers in high education (Liu, 2018).

In summary, students, teachers, and HR units are each a distinctive type of customer that possesses different characteristics and attributes. Students, as the most direct customers of higher education (defined as special customers to distinguish them from the HR units), are both customers and products. They pay tuition fees to purchase educational resources from the school and thus act as consumers or customers at this stage. After learning for some time, however, their status shifts from customers to products that are “consumed” by HR units. Teachers are the quintessential school employee, enjoying salaries, a work environment, and services such as personal development provided by the institution. Modern corporate management theory views them as internal customers of higher education. One perspective maintains that the satisfaction of internal customers is more crucial than that of external customers to corporate survival and development (Tao, 2008; Wu et al., 2022a,b). Finally, HR units are the most direct external customers of higher education. They receive the direct products—students—as employees that serve their units, whose efficiency and performance are directly affected by the quality of the graduate products. At the same time, the levels of satisfaction expressed by HR units has a direct bearing on student employment and is therefore also a critical indicator of HE quality. Figure 2 illustrates these relationships between students, teachers, and HR units.

## Materials and methods

### Research methodology

The research used three methods to build the satisfaction index system. A literature review established the dimensions and items to be considered when designing the self-assessment tool. Interviews with expert informants laid the foundation for preparing the questionnaire and confirming its items and dimensions. Piloted questionnaires with various participants enabled the satisfaction survey to be tested, adjusted, and finalized.

The procedures and statistical methods for constructing the satisfaction scale in this study are as follows:

(1) Establish evaluation index database through literature.
(2) Summarize, consolidate, and streamline index.
(3) Select indicators through expert interviews and questionnaire.
(4) Select indicators through critical value and total-item analysis.
(5) Modified scale constructs use EFA.
(6) Validate model use SEM.

### Research objects

The survey participants were students, teachers and staff, and employers. We recruited 1,056 students (736 men and 320 women), who completed the Survey for the Chinese University Student Satisfaction Index, the Chinese University Student Satisfaction Presurvey Scale, and the Preliminary Measurement Scale for Chinese University Student Satisfaction. We also surveyed 371 teachers and staff (220 men and 151 women) using the Survey for the Chinese University Teacher and Staff Satisfaction Index, the Chinese University Teacher and Staff Satisfaction Presurvey Scale, and the Preliminary Measurement Scale of Chinese University Teacher and Staff Satisfaction. Finally, 174 people employed in Chongqing City or Sichuan, Guizhou, Jiangxi, and Zhejiang provinces completed the Preliminary Measurement Scale of Employer Satisfaction for Chinese University Graduates (Draft Version), the Preliminary Measurement Scale of Employer Satisfaction for Chinese University Graduates, and the Survey of Employer Satisfaction for Chinese University Graduates.

The study also interviewed 53 experts (36 men and 17 women), including education experts, language experts and sports experts. And these experts come from various universities and education government departments in China (Table 1).

**TABLE 1:** The research objects information.

	Participants	Men	Women	Total
Questionnaire	Students	736	320	1,056
	Teachers and staff	220	151	371
	Employers	125	49	174
Interview	Experts	36	17	53
Total			1,654	

## Results

### The student satisfaction survey

#### Determining the initial items

The Survey for the Chinese University Student Satisfaction Index was combined with a literature review to obtain 319 key indices that were subsequently merged and reduced to yield 70 items by a team of satisfaction and HE research experts. Following further review of the literature, discussions with experts, and piloting of the survey, these indices were classified into the following seven constructs: “teaching and study,” “student management and coaching,” “logistics services,” “academic cultural activities,” “learning and scientific research environments,” “school reputation and development,” and “internship and employment.”

#### Confirming the preliminary scale

To ensure the comprehensiveness, clarity, and rationality of the items used to measure each construct, we emailed the 70 key indices to five psychology experts to evaluate the face validity, logical validity, and language used. This allowed us to transform the 70 indices into the Chinese University Student Satisfaction Presurvey Scale, which was then tested and reduced to 67 items in the preliminary survey scale, 16 of which were included in the “teaching and learning,” construct, 13 in “student management and coaching,” 11 in “logistics services,” 6 in “academic cultural activities,” 9 in “learning and scientific research environments,” 4 in “school reputation and development,” and 8 in “internship and employment.” Responses were recorded on a 7-point Likert scale (1 = strongly disagree; 7 = strongly agree).

#### Analysis of the preliminary scale

##### Reliability analysis

The Cronbach’s α values of the constructs in the preliminary survey scale were 0.967 for “teaching and learning,” 0.952 for “student management and coaching,” 0.925 for “logistics services,” 0.940 for “academic cultural activities,” 0.946 for “learning and scientific research environments,” 0.926 for “school reputation and development,” and 0.970 for “internship and employment,” with an overall Cronbach’s α for the scale of 0.987. All these values were all greater than 0.80, indicating that the scale was reliable overall and the reliability of each construct was satisfactory.

##### Critical value and total-item analysis

After calculating the total scores from the samples, a low-scoring group consisting of the lowest 27% of scores (total ≤ 281) and a high-scoring group (the highest 27%, total ≥ 395) were identified. An independent samples *t*-test was conducted for the two groups and demonstrated that, for all items, significant differences existed at a statistical level of *p* < 0.1. Therefore, the critical value differences in this study were all statistically significant, and no item was removed. The correlation analysis of all items revealed that the minimum correlation coefficient was 0.658, well above the 0.30 threshold and higher than any other-item correlation, so no item required omission.

##### Validity analysis of modified scale constructs

Principal axis factoring was adopted in this study, with exploratory factor analysis (EFA) performed *via* oblique rotation and using the standard Kaiser criterion for scale data that had passed critical value screening and total-item, other-item analysis.

Exploratory factor analysis was conducted three times in this study: the factor loadings were too low in the first two iterations and two-factor loadings were observed, which were then removed. The removal of the seventh factor left one construct containing only a single item, which was also removed following Wu (2000) recommendation that constructs with fewer than three items should be cut. Subsequently, EFA was conducted a third time (KMO = 0.972, Bartlett’s Sphericity Test *p* = 0.00). After oblique rotation, we discovered that six factors had eigenvalues larger than 1 (Table 2). The amount of variance each explained was 54.466, 4.677, 3.065, 2.545, 2.266, and 1.527%, and their cumulative contribution was 68.545%. For all constructs, all items had a factor loading of 0.3 or more.

**TABLE 2:** Total explained variance of the third exploratory factor analysis (EFA).

Construct	Initial eigenvalue			Extraction sums of squared loadings			Rotation sums of squared loadings
	Total	Variance %	Accumulated %	Total	Variance %	Accumulated%	Total
1	31.893	54.989	54.989	31.590	54.466	54.466	25.499
2	3.040	5.241	60.230	2.712	4.677	59.142	17.779
3	2.029	3.498	63.728	1.778	3.065	62.208	23.600
4	1.752	3.021	66.748	1.476	2.545	64.752	13.770
5	1.606	2.770	69.518	1.314	2.266	67.018	21.058
6	1.170	2.017	71.535	0.885	1.527	68.545	17.961

After rotation, the component matrix revealed that all 58 remaining items could be discretely categorized into six constructs. Constructs 1, 2, 3, 4, 5, and 6 contained 16, 11, 9, 6, 6, and 10 items, respectively. The names of the constructs were “teaching and learning,” “logistics services,” “internship and employment,” “learning and scientific research environments,” “academic culture and life,” and “student management and coaching.” After comparison with the preliminary test scale, the “school reputation and development” construct was deleted.

#### Hypothetical model of student satisfaction

Based on the item analysis results from the preliminary test scale, we hypothesized a second-order, seven-factor model of student satisfaction within a particular university. The first order contained teaching and learning, logistics services, internship and employment, learning and scientific research environments, and student management and coaching. The second order consisted of these six related first-order satisfaction constructs combined into one factor: student satisfaction.

#### Construct validity of the revised student satisfaction scale, based on exploratory factor analysis

##### Reliability analysis

The Cronbach’s α values of the constructs in the revised scale were 0.955, 0.908, 0.953, 0.927, 0.929, and 0.930, respectively. The Cronbach’s α of the overall scale was 0.979. Because the Cronbach’s α values of each construct and the overall scale were all greater than 0.80, the scale was considered entirely reliable The correlation coefficient of each construct was between 0.534 and 0.744, showing their strong interrelationships and relative independence from one another.

##### Construct validity analysis and verification of the hypothetical model

The item analysis retained 58 items in the formal scale to collect data for EFA using structural equation modeling software (AMOS 17.0). Maximum likelihood was used to estimate the model, and a covariance matrix was used. Raw data were obtained from a sample of 382 people. The large number of items was reduced to 18 in order to reduce the number required for processing.

The analysis showed that X2/DF = 2.66 (343.120/129) and *p* = 0.00 < 0.01, thereby falling within the 1–3 range proposed by Ho et al. (2004). The non-normed fit index (NNFI) was 0.968, the goodness of fit index (GFI) was 0.911, the adjusted goodness of fit index (AGFI) equaled 0.881, and the comparative fit index (CFI) was 0.973. All these indices reached or approached 0.90, the standard proposed by Finlay (1996). The root mean square error (RMSE) was 0.066, smaller than 0.08, thereby indicating an acceptable model fit (Finlay, 1996). Both the overall model fit and the scale’s construct validity were acceptable.

**FIGURE 3:**
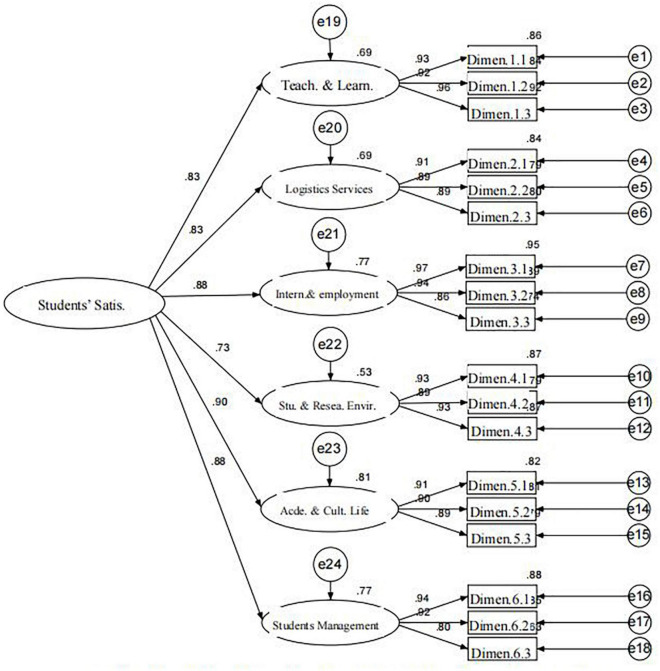
Standardized estimation model of Chinese University student satisfaction scale.

The standardized estimate model of the measurement model is shown in Figure 3. Each item (combined item) had relatively high factor loadings (>0.8) in their corresponding constructs. The factor loadings of the six constructs were 0.83, 0.83, 0.88, 0.73, 0.90, and 0.88 while their predictability values were 0.69, 0.69, 0.77, 0.53, 0.81, and 0.77, respectively. Figure 3 also revealed that the “academic culture and life” construct had the largest factor loading and the highest predictability in the student satisfaction survey.

#### Summary

In the first part of this study, we used software to compile an online survey and paper questionnaire that were distributed to 1,056 university students. After data analysis, the China University Student Satisfaction Scale was finalized. The scale comprised 58 items over 6 dimensions: “teaching and learning,” “logistics services,” “internship and employment,” “learning and scientific research environments,” and “student management and coaching.” The reliability and validity analyses and model validation all returned positive results. Furthermore, each index of the hypothetical model based on the scale achieved the required standard to confirm the goodness of fit.

### The teacher satisfaction survey

#### Confirming the initial items

The Survey for the Chinese University Teacher Satisfaction Index was used to conduct surveys and relevant literature analysis, with 187 key indices reduced into 58 items by an expert team consisting of five satisfaction researchers and three HE scholars. The literature review and survey results enabled all indices to be classified into the following eight constructs: “basic work situations,” “social acknowledgment,” “salary,” “logistics services,” “scientific research and management,” “relationships with each administrative level,” “a humanistic environment,” and “overall school development.”

#### Confirming the preliminary scale

To ensure that the items of each construct were comprehensive, clear, and valid, we emailed the 58 key indices to five psychology experts, who evaluated the face validity, logical validity, and language clarity of the items in the preliminary survey scales. The 58 key indices then became the Chinese University Teacher Satisfaction Presurvey Scale that was used for the presurvey. Based on the results and the experts’ opinions, the presurvey scale was further supplemented, screened, and modified. Two language experts were hired to further amend the clarity of the language used in the scale.

Based on the opinions of the experts and the teachers who were surveyed, the Preliminary Measurement Scale of Chinese University Teacher Satisfaction was modified to create three preliminary scales linked to our research aims: the Scales for Teachers of Professional Courses and General Education Courses (both of which consisted of eight constructs and fifty-five items), and the Scale for Administrators and Others (eight constructs and forty-five items). This study adopted a 7-point Likert scale (1 = strongly disagree; 7 = strongly agree).

#### Analysis of the preliminary scale

##### Reliability analysis

The Cronbach’s α values of the constructs in the preliminary survey scale were as follows: 0.944 for “basic work situations,” 0.927 for “social acknowledgment,” 0.924 for “salary,” 0.872 for “logistics services,” 0.941 for “scientific research and management,” 0.894 for “relationships with each administrative level,” 0.941 for “a humanistic environment,” and 0.953 for “overall school development.” The overall Cronbach’s α for the scale was 0.981. Because all these values were greater than 0.80, the survey can be considered sound and reliable.

##### Critical value and total-item analysis

After calculating the total scores from the samples, we isolated a low-scoring group (the lowest 27%, scoring ≤ 161) and a high-scoring group (the highest 27%, scoring ≥ 221). The independent samples *t*-test conducted for these groups showed they differed at a statistically significant level of *p* < 0.01. The minimum correlation coefficient for all items was 0.650, considerably more than 0.30 and larger than the other-item correlations, so all the items were retained.

##### Analysis of the modified scale’s construct validity

In the first three of the four EFAs conducted, two-factor loadings were observed, so these 16 items were removed. After the oblique rotation of the fourth EFA (KMO = 0.937, Bartlett’s Sphericity Test *p* = 0.00), four factors were found to have eigenvalues above 1 (see Table 3), explaining 51.939, 7.486, 4.736, and 3.077% of the variance (67.238% overall). All items in every construct had factor loadings of 0.3 or more.

**TABLE 3:** Total explained variance of the fourth exploratory factor analysis (EFA).

Construct	Initial eigenvalue			Extraction sums of squared loadings			Rotation sums of squared loadings
	Total	Variance %	Accumulated %	Total	Variance %	Accumulated %	Total
1	15.373	53.009	53.009	15.062	51.939	51.939	12.101
2	2.502	8.629	61.638	2.171	7.486	59.425	11.374
3	1.705	5.878	67.516	1.373	4.736	64.160	8.965
4	1.206	4.160	71.675	0.892	3.077	67.238	6.368

After rotation, the component matrix revealed that the remaining 29 items could be categorized into four constructs. Constructs 1, 2, 3, and 4 contained 9, 10, 6, and 4 items, respectively. Because, after item analysis, the constructs and items differed greatly from those in the preliminary scale, we renamed the four resulting constructs “school humanistic environment and development,” “basic work situation and social identity,” “salary and promotion,” and “logistics services.”

#### Hypothetical model of teacher and staff satisfaction

Through EFA and integration of the item analysis results from the preliminary test scale, a second-order five-factor model (second-order one-factor and first-order four-factor model) of teacher and staff satisfaction was developed. The first order consisted of “basic work situation and social identity,” “salary and promotion,” “logistics services,” and “school humanistic environment and development.” The second order comprised the four related first-order satisfaction constructs condensed into one factor: “teacher and staff satisfaction.”

#### Construct validity of the revised teacher and staff satisfaction scale, based on exploratory factor analysis

##### Reliability analysis

The Cronbach’s α value of the constructs in the revised scale (“basic work situation and social identity,” “salary and promotion,” “logistics services,” and “school humanistic environment and development”) were 0.922, 0.913, 0.959, and 0.935, respectively. The Cronbach’s α of the overall scale was 0.972. All these values demonstrated that the scale, constructs, and related indices provided satisfactory reliability and stability. The correlational coefficient of each construct was between 0.581 and 0.826, showing that each construct was closely linked to the others but was also relatively independent.

##### Construct validity analysis and verification of the hypothesized model

Through item analysis, 29 items were retained in the formal scale to collect EFA data using AMOS software (v. 17.0). Maximum likelihood estimation was used to develop the model and a covariance matrix was used. Raw data from a sample (*N* = 170) were inputted and the items in each construct were combined to obtain 12 new items.

The analysis demonstrated that X2/DF = 3.478 (173.892/50), *p* = 0.000 < 0.01, thereby meeting the threshold of X2/DF < 5. The additional values were as follows: NNFI = 0.934, GFI = 0.865, AGFI = 0.790, and CFI = 0.950, in line with the 0.90 threshold recommended by Stevens (1996). However, the RMSE value was 0.121, some distance from the acceptable standard of optimal model fit, possibly due to the small sample size. Nonetheless, we believe that the overall model fit was acceptable, as was the scale’s construct validity.

**FIGURE 4:**
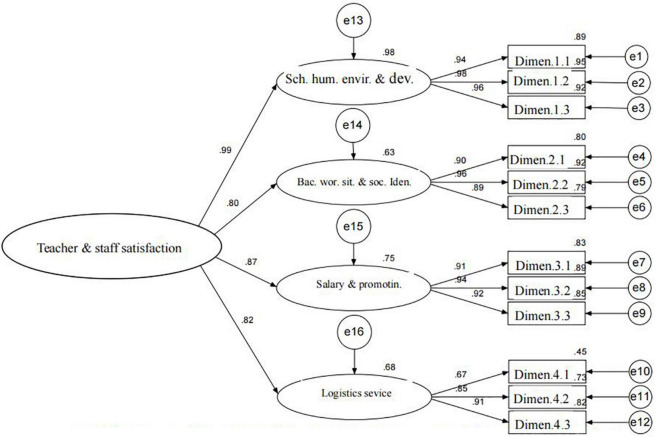
Standardized estimation model of Chinese University teacher and staff satisfaction scale.

The standardized estimation of the measurement model is provided in Figure 4. All items (combined) had relatively high factor loading values (>0.8) within their corresponding constructs. The loadings for “basic work situation and social identity,” “salary and promotion,” “logistics services,” and “humanistic school environment and development,” were 0.99, 0.80, 0.87, and 0.82, respectively, while the predictability values of the constructs were (in the same order) 0.98, 0.63, 0.75, and 0.68. Figure 4 also shows that the “humanistic school environment and development” construct had the largest factor loading and the highest predictive value in the teacher and staff satisfaction survey.

#### Summary

In this part of the study, an online and paper-based satisfaction survey was devised and distributed to 478 university teachers and staff. Following data analysis, the Chinese University Teacher and Staff Satisfaction Survey Scale was finalized to contain four dimensions: “basic work situation and social identity,” “salary and promotion,” “logistics services,” and “humanistic school environment and development,” and a total of twenty-nine items. The reliability and validity analyses and model validation returned favorable results and because each index of the hypothesized model based on the scale met the required standard, the model fit the data satisfactorily.

### Compilation of the human resources unit satisfaction survey

#### Interviews and confirming the preliminary items

Following interviews with HR managers, two graduate students independently extracted 124 key HR satisfaction indices. These were subsequently reduced to 48 items by the team of satisfaction researchers alongside two career counselors. Following further discussions with experts, an analytic review of literature, and piloting of the items with Chinese university HR units, the items were classified into the following seven constructs: “basic career literacy,” “basic skills,” “interpersonal relationships,” “implementation and innovation,” “adaptation and development,” “professional skills,” and “overall evaluation.”

#### Confirming the preliminary scale

To ensure that the items measuring each construct were comprehensive, clear, and reasonable, we emailed the 48 key indices to 5 psychology experts, who evaluated the face validity, logical validity, and linguistic clarity of the items in the preliminary scales. The resulting 48 items constituted the Chinese University Graduate Employer Satisfaction Presurvey Scale. Based on the results of the piloting and the experts’ opinions, the presurvey scale was further modified. Two language experts were asked to further amend the wording, resulting in the Preliminary Measurement Scale of Employer Satisfaction for Chinese University Graduates, which contained seven constructs and forty-six items. Constructs 1, 2, 3, 4, 5, 6, and 7 contained 11, 9, 6, 7, 6, 5, and 2 items, respectively. Responses were measured on a 7-point Likert scale (1 = “strongly disagree”; 7 = “strongly agree”).

#### Analysis of the preliminary scale

##### Reliability analysis

The Cronbach’s α values for the constructs were 0.951 for “basic career literacy,” 0.956 for “basic skills,” 0.963 for “interpersonal relationships,” 0.963 for “implementation and innovation,” 0.959 for “adaptation and development,” 0.939 for “professional skills,” and 0.960 for “overall evaluation.” The overall Cronbach’s α for the scale was 0.988. Because all these values were greater than 0.80, the reliability of the overall scale and each individual construct was satisfactory.

##### Critical value and total-item analysis

Based on the sample scores, we identified a low-scoring group (the bottom 27% of the sample, scoring ≤ 265) and a high-scoring group (the top 27%, scoring ≥ 303). The results of the independent samples *t*-test conducted for these two groups showed that the differences for all items were significant at *p* < 0.01. The minimum correlation coefficient for all items was 0.650, comfortably larger than the 0.30 threshold, so no item was removed on this basis. However, other-item analysis revealed that some other-item correlations (item 11 in Construct 1 and items 6, 7, and 8 in Construct 2) were larger than the all-item correlation. Consequently, the first three items were excluded from the scale and the last (8) was transferred to Construct 5.

##### Analysis of construct validity in the modified scale

The Cronbach’s α values of the constructs used in the modified survey were 0.946 for “basic career literacy,” 0.934 for “basic skills,” 0.961 for “interpersonal relationships,” 0.958 for “implementation and innovation,” 0.963 for “adaptation and development,” 0.934 for “professional skills,” and 0.957 for “overall evaluation,” all of which were greater than 0.80. The overall Cronbach’s α of the modified scale was 0.985. These results demonstrated a good level of reliability in the overall scale and each construct.

##### Analysis of the preliminary scale’s construct validity

Three rounds of EFA were conducted, with the first two of these producing two-factor loadings on 20 items, all of which were removed. After the oblique rotation of the third EFA (KMO = 0.929, Bartlett’s Sphericity Test *p* = 0.00), we discovered three factors with eigenvalues above 1, explaining 66.163, 6.672, and 4.686% of the variance individually and 77.521% cumulatively (see Table 4).

**TABLE 4:** Total explained variance of the third exploratory factor analysis (EFA).

Construct	Initial eigenvalue			Extraction sums of squared loadings			Rotation sums of squared loadings
	Total %	Variance %	Accumulated %	Total %	Variance %	Accumulated %	Total %
1	15.439	67.127	67.127	15.217	66.163	66.163	12.941
2	1.740	7.566	74.692	1.535	6.672	72.835	12.665
3	1.318	5.732	80.425	1.078	4.686	77.521	9.655

After rotation, the component matrix revealed that all 23 items could be discretely categorized into one of three constructs. Constructs 1, 2, and 3 contained 9, 10, and 4 items, respectively, with the constructs labeled “career literacy and interpersonal relationships,” “innovation and capability,” and “basic skills.”

#### Summary

This phase of the study compiled and distributed an online survey and paper questionnaires to 174 university HR managers. Intensive data analysis allowed us to finalize the University Graduate Human Resources Unit Satisfaction Survey Scale, consisting of 23 items over 3 dimensions: “career literacy and interpersonal relationships,” “innovation and capability,” and “basic skills.” Because the percentage of valid questionnaires from this survey was small, we were unable to formally verify the scale’s reliability, structural validity, and model fit. In this instance, the use of subjective self-report methods may have impacted the reliability of the questionnaire responses. Therefore, the precision and reasonableness of this scale are unverifiable; the validity of the removed items could not be ascertained through quantitative methods.

## Discussion

### Conclusion

This research was motivated by recent trends toward using customer-centered public management theories to understand organizational behavior and attitudes. We believe it constitutes a bold and innovative attempt to introduce corporate management theories into Chinese HE research. The resulting scales can be used to promote the sustainable development of Chinese universities and provide ideas for management reform among Chinese business units.

The research utilized interviews and developed three separate surveys that were tested on 1,654 respondents from a selected university, including students, teachers, and staff, and HR unit managers. The final survey tool was based on a rigorous scientific process of review, piloting, expert consultation, and statistical analysis, and is named the *Chinese University Stakeholder Satisfaction Survey*. This consisted of three subscales: the *China University Student Satisfaction Scale* (6 dimensions and 58 items), the *China University Teacher and Staff Satisfaction Scale* (4 dimensions and 29 items), and the *China University Graduate Human Resources Department Satisfaction Scale* (3 dimensions and 23 items). These tools reflect the overall purpose of the survey and provide valid measurements of their respective objects.

The stakeholder satisfaction tools we designed can be used as self-assessment tools by universities. In addition, the system requires assessment implementation activities to improve its comprehensiveness and the surveys will need to be adjusted as internal and external school environments continue to change.

### Theoretical discussion

In China, public universities are a particular type of business whose revenues and expenditures are closely monitored by the government. However, this management system is ill-equipped to cope with rapidly changing socioeconomic conditions. As a consequence, universities are likely to grow increasingly maladapted and less able to fulfill their basic tasks of cultivating talents, serving society, and conducting scientific research.

Since 2003, the Chinese government has started a large-scale evaluation of universities. Through nearly 20 years of development, the pure government evaluation has not been enough to promote the sustainable development of universities. The evaluation of China’s HE lacks third-party evaluation and self-assessment. The three evaluations complement each other and perform their respective duties, and promote the quality of higher education together. In particular, self-assessment has an important value in promoting the sustainable development of universities. Internal university quality guarantees and monitoring systems are also indispensable to maintaining high levels of quality.

The present study focused on the internal management and self-evaluation of universities in China, making innovative use of Western corporate management theories to explore the internal management of HE in the country. The study was ultimately motivated by the urgent need to remove the administrative constraints that have long restricted Chinese universities. Our perspective proactively introduced corporate management philosophies into Chinese universities and developed a way to guarantee the quality of education. In line with other Chinese scholars (e.g., Zhang and Zhao, 2021) we believe that China’s HE evaluation processes should refocus from outcomes onto processes. Meanwhile, the importance of establishing a culture of continuous improvement in HE is also being gradually emphasized by Chinese scholars (Zhou and Yuan, 2020; Shi, 2022). In summary, organizations must develop the capacity to complete their missions and goals. Additional research is required to further improve institutional competitiveness and complete the development of quality assurance systems within Chinese HE.

### Practical discussion

In a globalized economy, HE management should maintain the characteristics of individual institutions and develop models based on advanced corporate management theories such as customer-centered self-assessment. By focusing on internal and external university shareholder satisfaction assessments and models based on the assumption of continuous change within the sector, the longstanding emphasis that Chinese university self-assessment places on results can shift to processual models.

Quality assurance reform is central to improving Chinese university management systems. Customer-centered corporate management approaches should be used to reform current models of HE administration and establish a comprehensive HE self-assessment system. Our stakeholder satisfaction assessment tools can be directly used by university management to assess their organizations, identify and prioritize issues, and thus ensure high-quality development. We recommend that institutions adapt the surveys to their particular conditions within a culture of continuous evaluation and improvement. From our perspective, self-assessment is both an evaluative and a management process, a view shared by others working in the field (Liu, 2022). The use of modern corporate tools and ideas for university management is also crucial for promoting the quality of high school education services.

### Research limitations

There are some research limitations. First, there can be little doubt that the number of research participants (170) affected the structural validity of the Teacher Satisfaction Survey and the verification of the underlying hypotheses. The individual indices indicated that the model fit was only marginal. Additionally, resource constraints meant that some more sensitive indices (such as finance systems) were excluded from this study.

Second, we had to mail the questionnaire on HR satisfaction because the participants were widely dispersed. As a result, we had much less control over the completion process, lowering the quality of the retrieved questionnaires and reducing the overall retrieval rate, resulting in a small sample size that fulfilled the requirements for EFA but not for structural validity and verification of the full hypothetical model. Future efforts should aim to address the shortcomings linked to this subscale.

Finally, when developing the three surveys, we did not thoroughly consider how to optimize integrating modern management philosophy with Chinese higher education. That is to say, we failed to control for some subjective factors such as the effects of individual education on students’ growth in the student survey, the cultural specificity of university teachers in the staff survey, or, for the HR survey, the possibility that the graduates would continue to develop as products of higher education.

## Data availability statement

The original contributions presented in this study are included in the article/supplementary material, further inquiries can be directed to the corresponding author.

## Ethics statement

The studies involving human participants were reviewed and approved by the Ethics Committee of the Sichuan University. The participants provided their written informed consent to participate in this study.

## Author contributions

FP performed the initial analyses and wrote the manuscript. LL and ZW assisted in the data collection and data analysis. All authors revised and approved the submitted version of the manuscript.
